# Influence of vertical GPS sensor placement on criterion agreement and interchangeability of distance and speed measures

**DOI:** 10.1371/journal.pone.0357116

**Published:** 2026-09-10

**Authors:** Lee Rooney, Gerard McMahon, Rodney Kennedy

**Affiliations:** School of Sport and Exercise Sciences, Ulster University, Belfast, United Kingdom; Portugal Football School, Portuguese Football Federation, PORTUGAL

## Abstract

This study evaluated agreement between PlayerTek™ 10 Hz GPS-derived total-distance and peak-speed measurements and practical field-based criterion measures and examined whether vertical sensor placement influenced agreement characteristics and measurement interchangeability. Following quality-control procedures, 758 paired observations were retained for analysis. Total-distance analyses included 533 paired observations from 55 participants collected during a modified team-sport simulation circuit, while peak-speed analyses included 225 paired observations from 68 participants during linear sprint testing. GPS units were positioned simultaneously at upper-back and mid-back locations. Agreement and measurement variability were assessed using established agreement-based statistical procedures, with secondary analyses evaluating placement equivalence and interchangeability. The upper-back placement demonstrated superior agreement with criterion measures compared with the mid-back placement. For total distance, upper-back measurements exhibited a small bias (0.57 m) and low variability (CV = 1.54%), whereas the mid-back placement demonstrated substantial systematic underestimation (−12.69 m), greater variability (CV = 4.18%), and poor agreement between placements. Equivalence testing confirmed that upper-back and mid-back placements were not interchangeable for total distance. For peak speed, upper-back measurements demonstrated stronger agreement with the criterion measure (ICC(A,1) = 0.864; CCC = 0.864) than the mid-back placement (ICC(A,1) = 0.770; CCC = 0.769), with smaller bias and lower variability. GPS-derived total-distance and peak-speed measurements demonstrated superior agreement characteristics when sensors were positioned at the manufacturer-recommended upper-back location. A small 10 cm inferior displacement of the sensor substantially altered total-distance measurements and reduced agreement with criterion measures, whereas peak-speed measurements were less affected. These findings highlight the importance of standardised sensor placement within applied athlete-monitoring environments.

## Introduction

The quantification of external load has become a central component of performance monitoring in team sports, with wearable global positioning system (GPS) technology widely adopted to measure variables such as total distance and peak running speed. The widespread integration of GPS-derived metrics into applied sport reflects their perceived ability to provide objective, time-efficient information regarding player movement demands, training responses, and competitive workloads. Consequently, GPS-derived measures are routinely used to inform training prescription, monitor fatigue, evaluate performance, and support return-to-play decision-making across a range of team-sport environments [1–3].

Foundational investigations reported that GPS-derived distance measurements generally fell within acceptable error margins under both controlled and sport-specific conditions [4–6]. Subsequent advances in satellite technology, signal processing, and sampling frequency have further improved measurement performance, with modern 10 Hz GPS units demonstrating enhanced validity and reliability for both distance- and speed-related variables [7–9]. Despite these improvements, measurement accuracy remains dependent on movement characteristics, with greater error typically observed during high-speed running, rapid accelerations, decelerations, and complex multidirectional movements [10–12].

From a practical perspective, athlete-tracking technologies are frequently implemented in applied environments before their measurement properties and practical utility are fully established for all performance variables of interest [13]. Recent systematic reviews have highlighted substantial variability in reported validity and reliability outcomes across GPS manufacturers, device generations, movement conditions, and analytical approaches [12,14]. Such variability suggests that methodological factors beyond device specifications alone may influence measurement outcomes and should be considered when evaluating athlete-monitoring technologies. Furthermore, the usefulness of any athlete-monitoring system is dependent not only on its agreement with a criterion measure but also on the consistency and reproducibility of measurements obtained under repeated conditions, as measurement variability may influence the interpretation of longitudinal changes in athlete performance and training load [15,16].

An additional and comparatively underexplored source of potential measurement error is sensor placement. Most GPS validation studies position devices at the manufacturer-recommended location between the scapulae and implicitly assume that this placement provides representative measurements of whole-body movement. However, evidence from wearable-sensor and inertial measurement unit (IMU) research suggests that recorded signals are sensitive to anatomical location. Previous investigations have demonstrated that accelerometer-derived load metrics vary with sensor position [17] and that trunk-mounted sensor outputs are influenced by the nature and magnitude of the movements being measured [18–20]. Collectively, these findings suggest that sensor-derived metrics may be sensitive to both movement characteristics and device placement. More recently, the importance of device placement has also been highlighted in evaluations of the reliability of athlete-monitoring systems [21].

The potential influence of sensor placement is particularly relevant because modern athlete-monitoring devices integrate GPS and inertial sensing technologies within a single wearable unit. Consequently, movement artefacts associated with sensor location may influence both accelerometer-derived and GPS-derived outputs. Recent GPS-specific investigations have begun to explore this issue. Terziotti et al. [22] reported device-dependent differences in displacement and energetic variables when multiple GPS units were worn simultaneously, whereas Akyildiz et al. [23] demonstrated that unit positioning may influence GPS-derived distance and maximal-speed measurements. Collectively, these findings suggest that anatomical placement may represent a meaningful source of measurement variability. However, few studies have simultaneously evaluated criterion agreement, measurement variability, equivalence, and interchangeability following relatively small changes in vertical GPS sensor placement using practical field-based reference measures. Consequently, the extent to which placement-related differences influence the interpretation of athlete-monitoring data remains unclear.

In applied athlete-monitoring environments, maintaining consistent sensor placement may be challenging across athletes, sessions, practitioners, and organisations. Furthermore, longitudinal datasets collected across multiple seasons, teams, or organisations may be vulnerable to subtle differences in device placement that are rarely documented or controlled. Given that athlete-tracking outputs are known to be influenced by methodological factors beyond device specifications alone, even relatively small variations in sensor location may influence the comparability of athlete-monitoring data and the interpretation of longitudinal performance trends [12,21].

From a methodological perspective, the evaluation of athlete-tracking technologies requires consideration of both the criterion measure employed and the statistical approach used to assess measurement properties. It is increasingly recognised that validity outcomes may vary according to the chosen reference system and the variable being assessed [24,25]. Furthermore, measurement-system selection itself may influence the magnitude of observed performance metrics, with differences between GPS- and ultra-wideband-based tracking systems producing practically meaningful discrepancies in tactical and positional analyses [26]. Consequently, agreement-based approaches such as Bland–Altman analysis are recommended to quantify systematic bias and limits of agreement rather than relying solely on correlation coefficients [15,27]. Similarly, concordance-based statistics provide a more appropriate assessment of agreement and interchangeability than traditional measures of association when evaluating competing measurement systems [28,29]. Importantly, validity, reliability, agreement, and interchangeability represent related but distinct measurement properties and should therefore be interpreted independently within athlete-monitoring research [16,29]. Recent evidence has further demonstrated that measurements obtained from different athlete-monitoring systems may exhibit varying degrees of practical equivalence, reinforcing the importance of explicitly evaluating interchangeability rather than assuming that agreement alone is sufficient [30]. Consequently, formal evaluation of interchangeability provides additional insight beyond conventional agreement statistics when determining whether measurements obtained under different methodological conditions can be used interchangeably [29,30].

It is also plausible that the influence of sensor placement differs according to the variable being assessed, as cumulative locomotor metrics and instantaneous velocity metrics are derived using different processing approaches. Cumulative locomotor metrics such as total distance and instantaneous velocity metrics such as peak speed are generated using different processing algorithms and may respond differently to changes in sensor orientation, movement artefact, and positional smoothing [7,8,11]. Consequently, the effects of sensor placement may not be uniform across GPS-derived variables, highlighting the need for metric-specific evaluation of measurement properties.

Despite the widespread adoption of PlayerTek/Catapult One systems within applied sport environments, relatively little independent evidence exists regarding the influence of sensor placement on agreement with practical reference measures.

Therefore, the primary aim of this study was to evaluate agreement between PlayerTek 10 Hz GPS-derived total distance and peak speed measurements and practical field-based reference measures (calibrated trundle wheel and 1080 Sprint). A secondary aim was to determine whether relatively small changes in vertical sensor placement influence agreement characteristics and placement interchangeability. Specifically, the study assessed: (1) agreement between GPS-derived total distance and a calibrated trundle-wheel reference measure; (2) agreement between GPS-derived peak speed and a 1080 Sprint device; and (3) the effects of upper-back and mid-back sensor placement on agreement, variability, and interchangeability. It was hypothesised that measurements obtained from the manufacturer-recommended upper-back placement would demonstrate stronger agreement with criterion measures than those obtained from a lower thoracic placement.

## Methods

### Participants

A total of 69 physically active team-sport athletes (60 male, 9 female; age 21.1 ± 1.3 years; stature 1.79 ± 0.07 m; body mass 77.5 ± 10.0 kg) volunteered to participate in this study. Participants were recruited from university-level sport programmes and included collegiate athletes with previous experience of high-intensity running, sprinting, and change-of-direction activities. Following data screening and application of variable-specific inclusion criteria, 55 participants contributed valid observations to the total-distance analyses, and 68 participants contributed valid observations to the peak-speed analyses. All participants were free from injury at the time of testing. Participant recruitment and data collection were conducted prospectively between June 2018 and February 2026. Ethical approval for this study was granted by the Ulster University Research Ethics Committee. All participants provided written informed consent prior to participation, and all study procedures were conducted in accordance with the principles of the Declaration of Helsinki.

### Experimental design

A repeated-measures agreement design was employed to evaluate agreement between GPS-derived measurements and practical field-based criterion measures and to determine the influence of vertical sensor placement on measurement characteristics. Two independent testing protocols were conducted: (i) a team-sport simulation circuit (TSSC) to assess total distance and (ii) maximal linear sprint testing to assess peak running speed.

**Fig 1:**
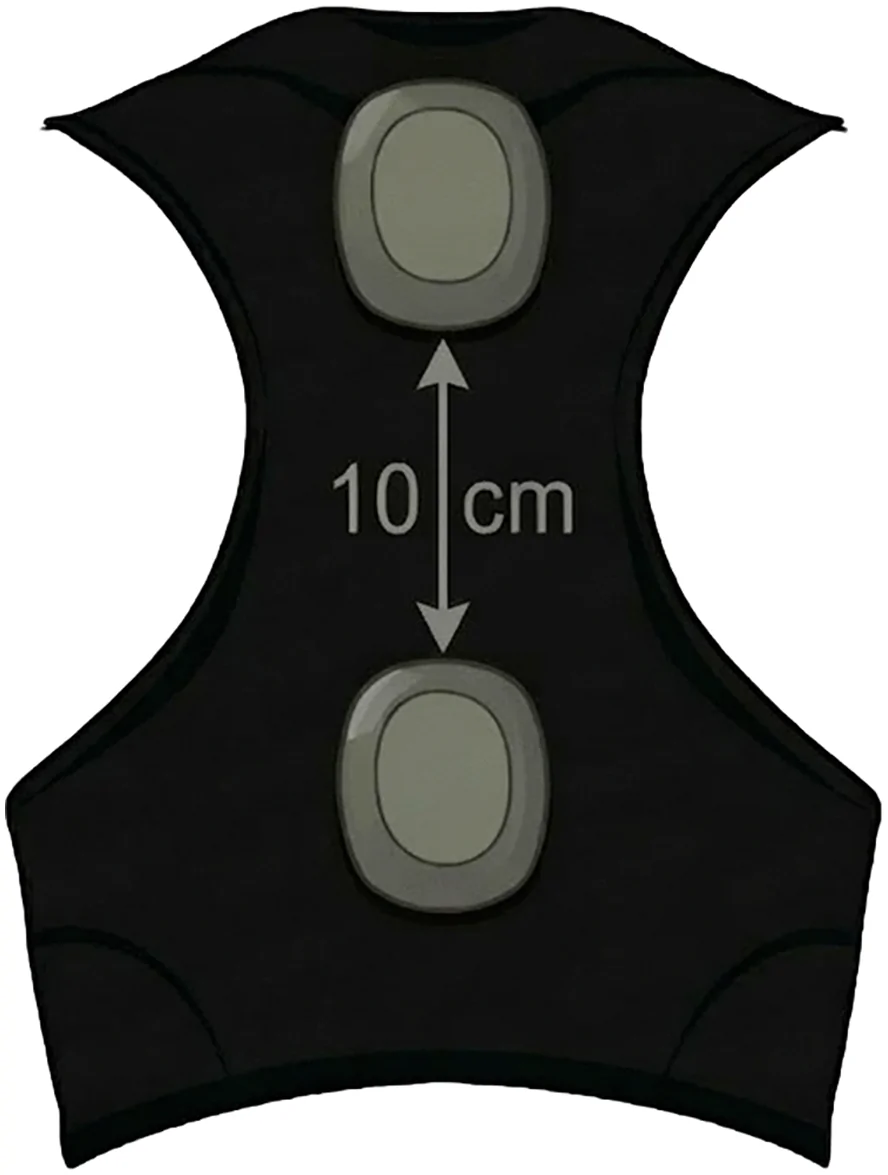
Placement of GPS units used in the present study. Two GPS units were positioned simultaneously at the upper-back (manufacturer-recommended location between the scapulae) and mid-back (10 cm inferior) using a fitted harness, enabling within-subject comparison of sensor-placement effects on measurement agreement.

During all testing procedures, participants simultaneously wore two GPS units positioned at different anatomical locations: (i) the upper back (manufacturer-recommended placement between the scapulae) and (ii) the mid-back (10 cm inferior) (Fig 1). This within-subject paired design enabled direct assessment of sensor-placement effects while controlling for inter-individual variability. GPS-derived outputs from both placements were compared against criterion measures using agreement-based analytical approaches consistent with current recommendations for evaluating athlete-monitoring technologies [1,24].

### Team-sport simulation circuit (Distance assessment)

**Fig 2:**
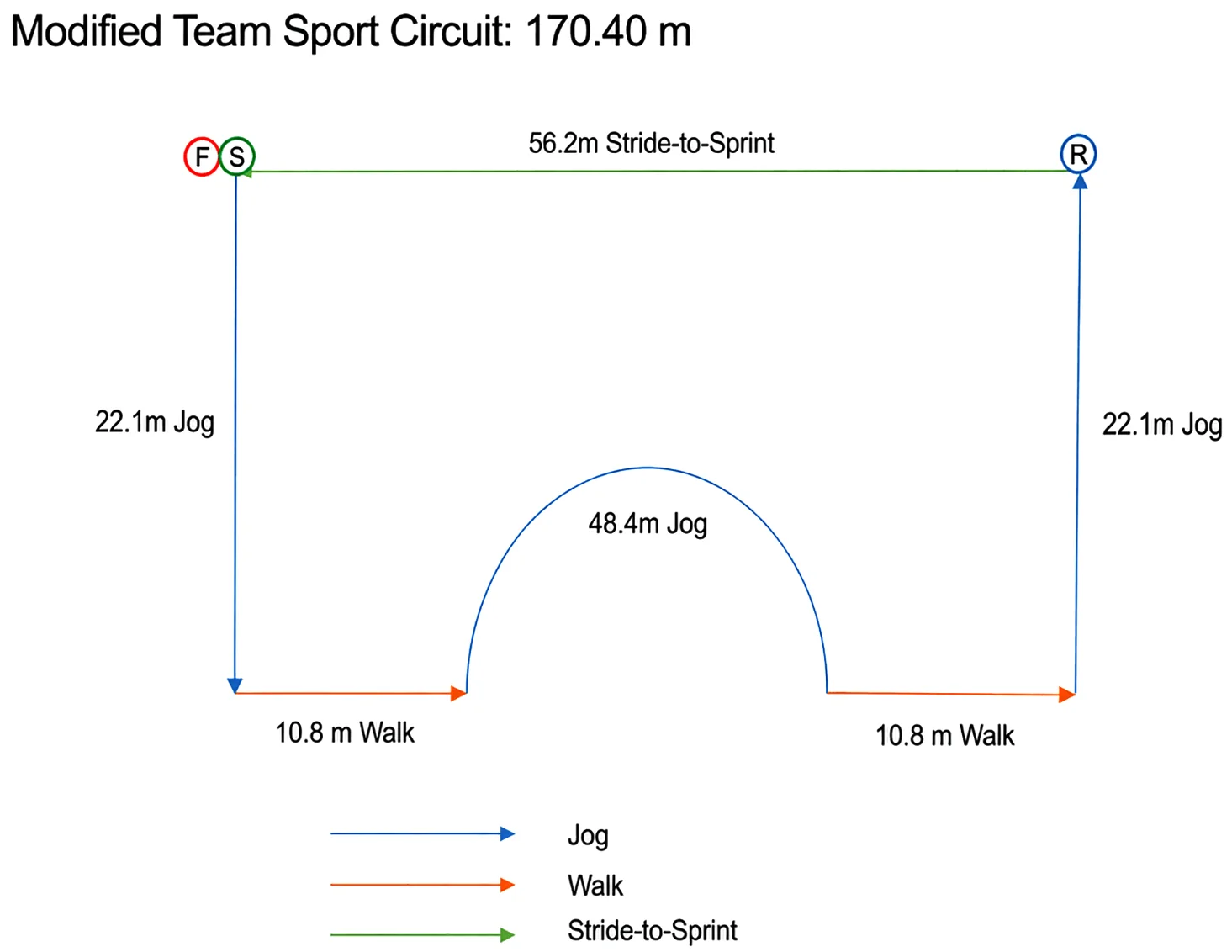
Schematic representation of the modified team-sport simulation circuit (TSSC; total distance = 170.40 m) used for total-distance assessment. The circuit incorporated segments of walking, jogging, stride-to-sprint running, and changes of direction representative of intermittent team-sport movement.

Total distance was assessed using a modified team-sport simulation circuit (TSSC) designed to replicate the intermittent locomotor demands of field-based team sports (Fig 2). The circuit incorporated walking, jogging, running, and changes of direction performed over a predefined course. Total circuit distance was 170.40 m, measured using a calibrated trundle wheel prior to testing and used as the fixed criterion reference distance throughout the study.

Participants completed multiple laps of the circuit under standardised conditions. Participants followed prescribed movement intensities using standardised verbal instructions, with consistent encouragement provided to ensure adherence to pacing requirements. The inclusion of sport-specific intermittent movement patterns aligns with previous GPS validation research [4,5,31].

### Linear sprint testing (Peak speed assessment)

**Fig 3:**
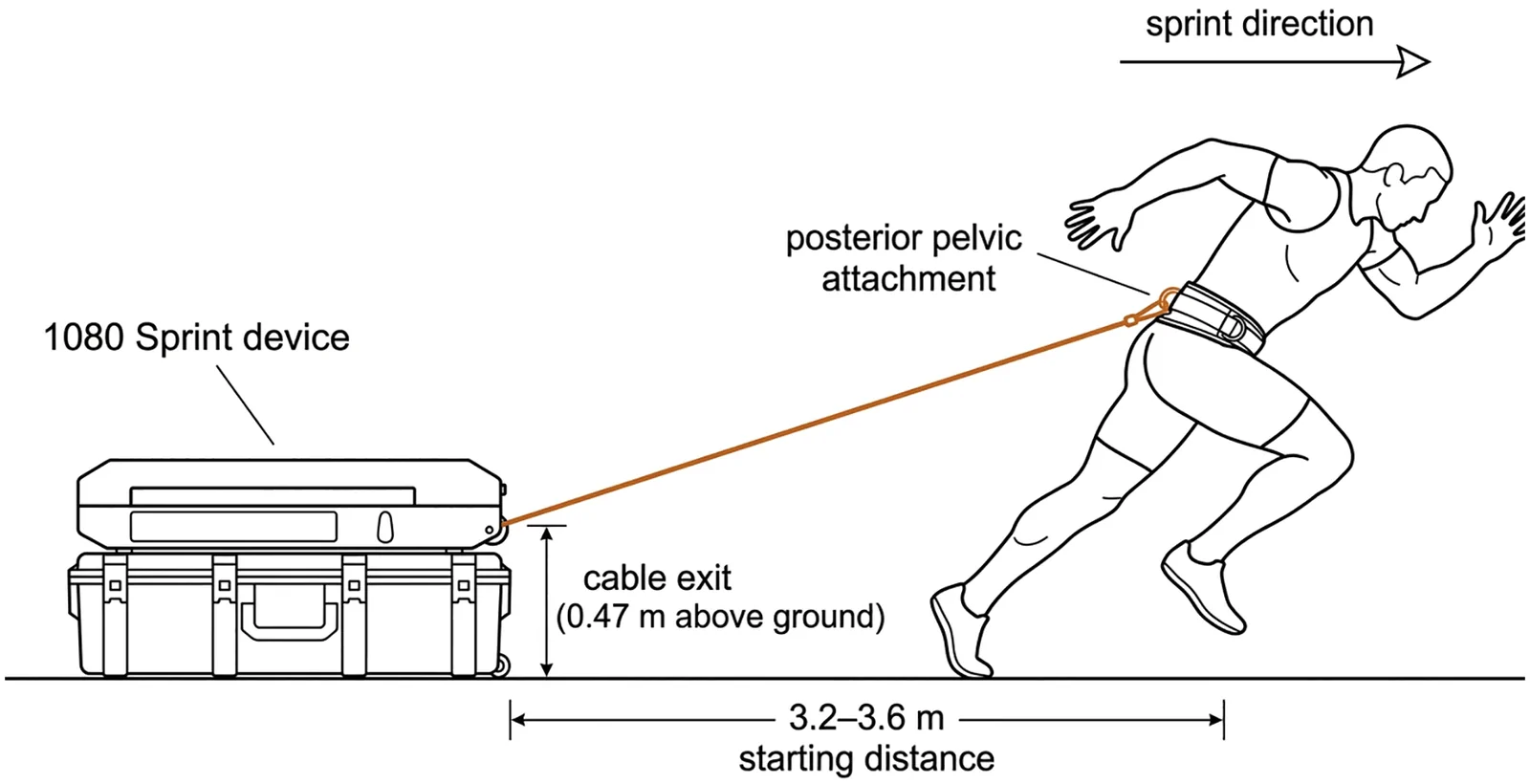
Schematic representation of the tethered sprint testing setup using the 1080 Sprint device. The tether was attached to a waist harness at the posterior pelvis while participants sprinted away from the device to assess peak sprint speed.

Peak running speed was assessed during maximal linear sprint efforts. Criterion measurements were obtained using a 1080 Sprint device (1080 Motion, Stockholm, Sweden), which provides high-resolution velocity measurements through a tethered cable system attached at the posterior pelvis (Fig 3). Previous research has demonstrated strong validity and reliability of the 1080 Sprint system for assessing sprint-performance variables, supporting its use as a practical reference measure for field-based velocity assessment [32].

Participants completed multiple maximal sprint trials separated by 2–3 min of passive recovery to minimise fatigue. Standardised verbal encouragement was provided during all trials. Peak speed was defined as the highest instantaneous velocity recorded during each sprint effort. The use of maximal linear sprint protocols is consistent with established methodologies for evaluating GPS-derived velocity measures [7,8].

### GPS instrumentation and sensor placement

Movement data were recorded using 10 Hz PlayerTek™ GPS units (Catapult Sports, Melbourne, Australia), now commercially marketed as Catapult One. The units incorporated global navigation satellite system (GNSS) receivers and integrated inertial sensors. GPS-derived total distance and peak speed were obtained using manufacturer-processed outputs within the PlayerTek software environment, reflecting typical applied athlete-monitoring practice. The same GPS device model, software environment, testing procedures, and criterion measurement methodologies were used throughout the study period.

Participants simultaneously wore two GPS units housed within manufacturer-provided harnesses. One unit was positioned at the upper back between the scapulae, consistent with manufacturer recommendations, while the second unit was positioned approximately 10 cm inferior at the mid-back (Fig 1). The vertical separation was selected to evaluate whether relatively small deviations from recommended placement influenced measurement agreement.

All devices were activated 10–15 min prior to testing to ensure satellite acquisition and signal stabilisation. Where possible, participants wore the same units throughout testing to minimise inter-unit variability, consistent with established recommendations [8,33,34]. Unit-allocation procedures were standardised throughout testing, with devices assigned consistently to upper-back and mid-back locations according to the study protocol. Simultaneous measurements were obtained from both placements during each trial, ensuring that between-placement comparisons were conducted under identical movement and environmental conditions. Satellite count and dilution-of-precision (DOP) metrics were not available within the PlayerTek software environment and therefore could not be reported.

### Data processing

GPS-derived total-distance and peak-speed data were exported directly from the manufacturer software without additional filtering or smoothing procedures. Criterion and GPS datasets were screened using predefined quality-control procedures.

A total of 761 paired observations were collected across both testing protocols. Observations were excluded only where technical or procedural issues were identified, including GPS signal dropout, device malfunction, criterion-device error, timing mismatch, or protocol deviation. Exclusions were applied identically across placement conditions. Following screening, three observations were removed, resulting in a final analytical dataset comprising 758 paired observations (S3 Dataset).

For the total-distance analyses, 533 valid paired observations from 55 participants were retained and analysed on a per-lap basis. For the peak-speed analyses, 225 valid paired observations from 68 participants were retained and analysed on a per-trial basis.

### Statistical analysis

Agreement between GPS-derived and criterion measurements was assessed using established agreement-based statistical procedures. Systematic bias and 95% limits of agreement (LoA) were evaluated using Bland–Altman analysis [27]. Bias was calculated as the average difference between methods, and LoA were calculated as bias ± 1.96 standard deviations of the differences. Bland–Altman plots were visually inspected to assess agreement patterns and identify potential systematic trends across the measurement range.

Agreement was quantified using complementary measures of concordance, reliability, and measurement error. These included Lin's concordance correlation coefficient (CCC), which evaluates both precision and accuracy between measurement methods [28], and the intraclass correlation coefficients (ICC(A,1)), which provide estimates of absolute agreement between measurements or measurement methods. For total-distance comparisons against the criterion measure, ICC(A,1) and CCC were not calculated because the reference distance remained constant (170.40 m) across all observations. As variance-dependent agreement statistics require variability in both methods, these indices are not estimable or informative when one method is invariant [15,29]. Under these conditions, agreement was interpreted primarily using Bland–Altman bias, limits of agreement (LoA), typical error (TE), and coefficient of variation (CV), as these metrics remain appropriate for evaluating agreement against a fixed-reference criterion.

Measurement variability was quantified using typical error (TE) and coefficient of variation (CV). Agreement analyses were performed for six predefined comparisons: (i) upper-back versus criterion distance, (ii) mid-back versus criterion distance, (iii) upper-back versus mid-back distance, (iv) upper-back versus criterion peak speed, (v) mid-back versus criterion peak speed, and (vi) upper-back versus mid-back peak speed.

Secondary analyses were conducted to evaluate placement interchangeability and equivalence. Interchangeability between upper-back and mid-back placements was assessed using the two one-sided tests (TOST) procedure applied to participant-level mean values [35]. Equivalence bounds were specified a priori as ±5.0 m for total distance and ±0.20 m·s^-1^ for peak speed, with sensitivity analyses performed using ±8.5 m and ±0.10 m·s^-1^, respectively. Equivalence was concluded when the entire 90% confidence interval for the mean between-placement difference fell within the predefined equivalence bounds.

Additional variability analyses were performed using participant-level absolute errors relative to the criterion measures. Mean paired differences in absolute error between placements were estimated using bootstrap procedures with 10,000 resamples and corresponding 95% confidence intervals (CI) were calculated. Error ratios were calculated as the mean absolute error of the mid-back placement divided by that of the upper-back placement.

Given the repeated-measures structure of the dataset, participant-level sensitivity analyses were undertaken to assess whether the primary findings were robust to clustering of repeated observations within individuals. Repeated observations were aggregated to participant-level mean values prior to analysis, and the agreement statistics were recalculated using these aggregated data. The participant-level findings were then compared with the primary observation-level analyses to determine whether the magnitude and direction of the observed placement effects were maintained following participant-level aggregation (S2 Text).

All analyses were conducted using Python (version 3.13). Agreement-based approaches were prioritised because correlation coefficients alone do not adequately assess systematic bias, agreement, equivalence, or interchangeability between measurement methods [15]. Statistical procedures were consistent with contemporary recommendations for evaluating measurement properties, agreement, equivalence, and interchangeability in athlete-monitoring research [24,29,30].

## Results

### Data screening and retention

A total of 761 paired observations were collected across the total-distance and peak-speed testing protocols. Following application of predefined quality-control procedures, three observations were excluded due to technical or procedural issues, resulting in a final analytical dataset comprising 758 paired observations.

For the total-distance analyses, 533 valid paired observations from 55 participants were retained and analysed on a per-lap basis. For the peak-speed analyses, 225 valid paired observations from 68 participants were retained and analysed on a per-trial basis. Simultaneous upper-back and mid-back GPS measurements were available for all retained observations, permitting direct between-placement comparisons.

### Descriptive statistics

Descriptive statistics and agreement metrics for GPS-derived total distance and peak speed are presented in Tables 1 and 2. Across both variables, measurements obtained from the upper-back placement demonstrated closer agreement with criterion measures than those obtained from the mid-back placement. Differences between placements were particularly evident for total-distance measurements.

**Table 1:** Agreement statistics for GPS-derived total-distance measurements relative to criterion measures and between sensor placements.

Comparison	Bias (m)	95% LoA (m)	TE (m)	CV (%)	ICC(A,1)	CCC
Upper-back vs Criterion	0.57	−6.74 to 7.87	2.64	1.54	NA	NA
Mid-back vs Criterion	−12.69	−31.70 to 6.32	6.86	4.18	NA	NA
Upper-back vs Mid-back	13.26	−4.57 to 31.09	6.43	3.91	0.089	0.089

Note. Bias = mean difference between methods. LoA = 95% limits of agreement. TE = typical error. CV = coefficient of variation. CCC = Lin's concordance correlation coefficient. ICC(A,1) = intraclass correlation coefficient. Criterion distance was fixed at 170.40 m as measured using a calibrated trundle wheel. ICC(A,1) and CCC were not estimable (NA) for criterion-distance comparisons because the criterion measure was invariant across all observations, rendering variance-dependent agreement statistics inappropriate.

**Table 2:** Agreement statistics for GPS-derived peak-speed measurements relative to criterion measures and between sensor placements.

Comparison	Reference Mean ± SD (m·s^-1^)	GPS Mean ± SD (m·s^-1^)	Bias (m·s^-1^)	95% LoA (m·s^-1^)	TE (m·s^-1^)	CV (%)	ICC(A,1)	CCC
Upper-back vs Criterion	7.89 ± 0.95	7.78 ± 0.83	−0.11	−1.00 to 0.77	0.32	4.09	0.864	0.864
Mid-back vs Criterion	7.89 ± 0.95	7.61 ± 0.88	−0.27	−1.40 to 0.85	0.40	5.22	0.770	0.769
Upper-back vs Mid-back	7.61 ± 0.88	7.78 ± 0.83	0.16	−0.59 to 0.91	0.27	3.50	0.885	0.885

Note. Bias = mean difference between methods. LoA = 95% limits of agreement. TE = typical error. CV = coefficient of variation. CCC = Lin’s concordance correlation coefficient. ICC(A,1) = intraclass correlation coefficient. Positive between-placement bias values indicate larger peak-speed measurements obtained from the upper-back placement. Criterion peak-speed values were obtained using the 1080 Sprint device.

### Agreement between GPS-derived and criterion total-distance measurements

**Fig 4:**
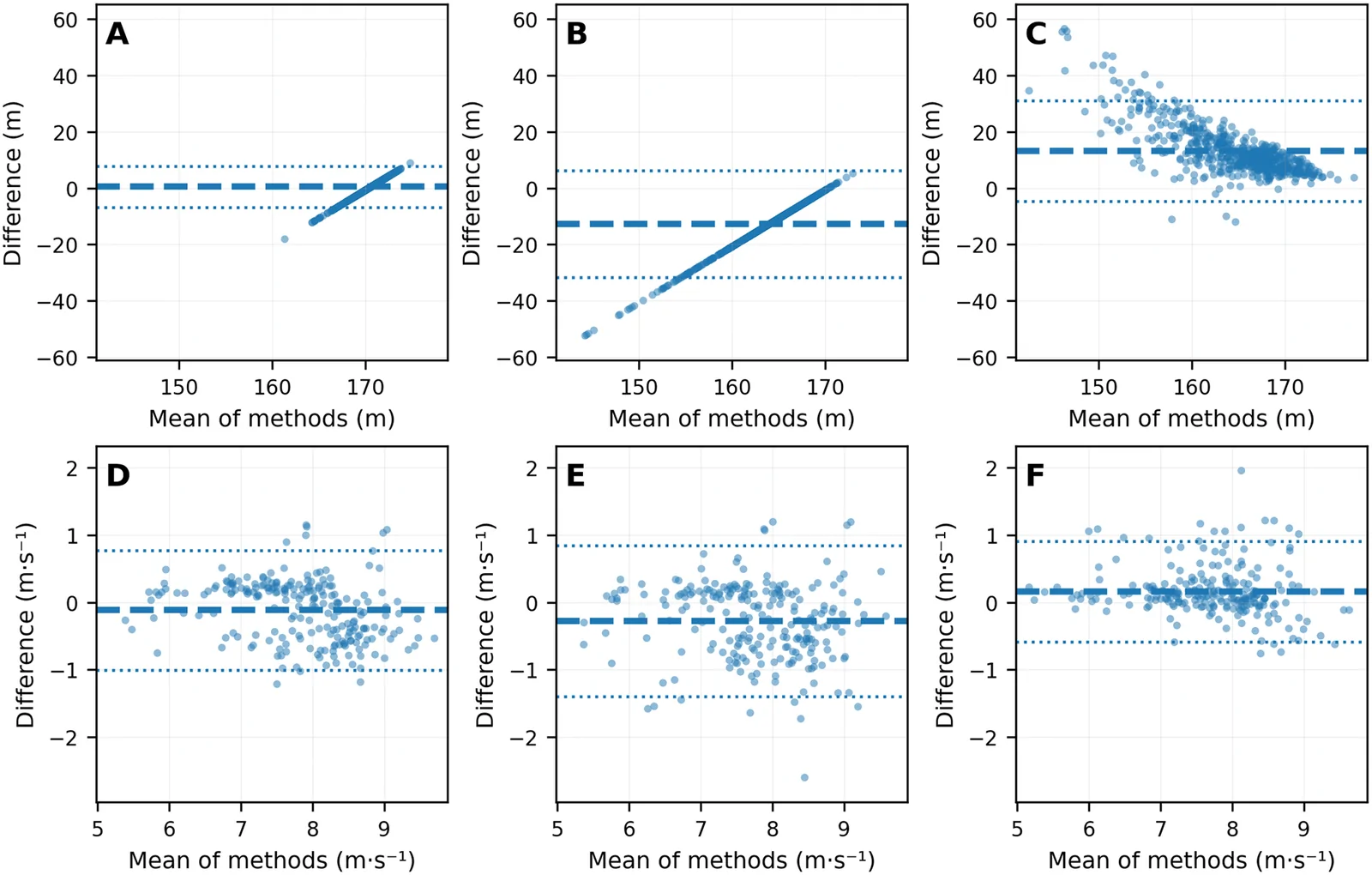
Bland–Altman analyses of GPS-derived total-distance and peak-speed measurements by sensor placement.

Agreement statistics for total distance are presented in Table 1 and Fig 4 (Panels A–C). ICC(A,1) and CCC were not calculated for criterion-distance comparisons because the criterion value was fixed across observations, rendering variance-dependent agreement statistics inappropriate.

The upper-back GPS placement demonstrated close agreement with the criterion distance measure, with a small positive bias of 0.57 m and relatively narrow limits of agreement (−6.74 to 7.87 m). Measurement variability was low (TE = 2.64 m; CV = 1.54%), indicating that total-distance measurements obtained from the manufacturer-recommended placement closely reflected the known circuit distance.

In contrast, the mid-back placement demonstrated substantially poorer agreement with the criterion measure. Mid-back measurements systematically underestimated total distance, producing a mean bias of −12.69 m with considerably wider limits of agreement (−31.70 to 6.32 m). Measurement variability was also substantially greater than that observed for the upper-back placement (TE = 6.86 m; CV = 4.18%).

Direct comparison between placements revealed a pronounced placement-dependent effect. The upper-back placement produced larger total-distance values than the mid-back placement, resulting in a mean between-placement bias of 13.26 m and wide limits of agreement (−4.57 to 31.09 m). Agreement between placements was poor (ICC(A,1) = 0.089; CCC = 0.089). Subsequent equivalence analyses confirmed that the two placement locations should not be considered interchangeable for total-distance assessment.

Visual inspection of the Bland–Altman plots (Fig 4, Panels A–C) supported these findings. The upper-back placement demonstrated close agreement with the criterion measure across the observed range, whereas the mid-back placement exhibited systematic underestimation. The between-placement analysis demonstrated a clear placement-dependent bias, with larger differences observed at lower mean values and progressively smaller differences at higher values.

### Agreement between GPS-derived and criterion peak-speed measurements

Agreement statistics for peak speed are presented in Table 2 and Fig 4 (Panels D–F).

The upper-back placement demonstrated close agreement with criterion peak-speed measurements, with a small negative bias of −0.11 m·s^-1^ and limits of agreement ranging from −1.00 to 0.77 m·s^-1^. Measurement variability remained low (TE = 0.32 m·s^-1^; CV = 4.09%), and agreement statistics indicated strong concordance between methods (ICC(A,1) = 0.864; CCC = 0.864).

The mid-back placement demonstrated a larger negative bias (−0.27 m·s^-1^), wider limits of agreement (−1.40 to 0.85 m·s^-1^), and greater measurement variability (TE = 0.40 m·s^-1^; CV = 5.22%) than the upper-back placement. Agreement with the criterion measure was correspondingly lower (ICC(A,1) = 0.770; CCC = 0.769).

Comparison of the two placements demonstrated a smaller placement effect for peak speed than for total distance. The upper-back placement produced slightly greater peak-speed values than the mid-back placement, resulting in a mean between-placement bias of 0.16 m·s^-1^. Agreement between placements remained comparatively high (ICC(A,1) = 0.885; CCC = 0.885), although the observed bias and limits of agreement indicate that placement effects were not completely negligible.

Bland–Altman plots (Fig 4, Panels D–F) demonstrated greater dispersion at higher running speeds, particularly for the mid-back placement. The between-placement analysis showed relatively small average differences but increasing variability as mean sprint velocity increased, suggesting reduced consistency between placements during higher-speed locomotor activity.

Panels A and D show agreement between the upper-back GPS placement and the criterion measures for total distance and peak speed, respectively. Panels B and E show agreement between the mid-back GPS placement and the criterion measures. Panels C and F show between-placement agreement (upper-back minus mid-back) for total distance and peak speed, respectively. Thick dashed horizontal lines represent mean bias, while dotted horizontal lines represent the 95% limits of agreement (LoA; ± 1.96 SD). Positive values indicate larger measurements obtained from the upper-back placement. The near-linear structure observed in Panels A and B reflects the fixed criterion distance used during testing, whereas Panel C illustrates the systematic placement-dependent differences between upper-back and mid-back measurements.

### Secondary analyses: Equivalence testing

Participant-level equivalence testing was conducted to evaluate whether upper-back and mid-back GPS placements could be considered interchangeable for total distance and peak speed (Table 3).

**Table 3:** Participant-level equivalence testing (TOST) of upper-back and mid-back GPS placements for total distance and peak speed.

Variable	Mean Difference	90% CI	Equivalence Bound	Equivalent
Total Distance	13.49 m	11.93–15.05 m	±5.0 m	No
Total Distance	13.49 m	11.93–15.05 m	±8.5 m	No
Peak Speed	0.142 m·s^-1^	0.092–0.193 m·s^-1^	±0.10 m·s^-1^	No
Peak Speed	0.142 m·s^-1^	0.092–0.193 m·s^-1^	±0.20 m·s^-1^	Yes

Note. Participant-level two one-sided tests (TOST) were performed using participant-level mean values. Mean differences are reported as upper-back minus mid-back values. Equivalence was concluded when the entire 90% confidence interval fell within the predefined equivalence bounds. Primary equivalence bounds were ±5.0 m for total distance and ±0.20 m·s^-1^ for peak speed; ± 8.5 m and ±0.10 m·s^-1^ were included as sensitivity analyses.

For total distance, equivalence was not supported under either the primary (±5.0 m) or sensitivity (±8.5 m) equivalence bounds. The mean between-placement difference was 13.49 m (90% CI: 11.93 to 15.05 m), with the entire confidence interval exceeding both equivalence thresholds. These findings indicate that upper-back and mid-back placements should not be considered interchangeable for total-distance assessment.

For peak speed, equivalence outcomes were dependent on the selected threshold. The mean between-placement difference was 0.142 m·s^-1^ (90% CI: 0.092 to 0.193 m·s^-1^). Equivalence was demonstrated under the practical ±0.20 m·s^-1^ bound but not under the more stringent ±0.10 m·s^-1^ bound, indicating that placement-related differences in peak speed were comparatively small but not negligible.

Variability comparisons based on participant-level absolute errors relative to the criterion measures demonstrated greater measurement error for the mid-back placement than the upper-back placement for both total distance and peak speed (S1 Text).

### Participant-level sensitivity analyses

Participant-level sensitivity analyses yielded agreement estimates that were similar in magnitude and direction to the primary observation-level analyses (S2 Text). Across both total distance and peak speed, the upper-back placement remained in closer agreement with the criterion measures than the mid-back placement. The direction and magnitude of the observed placement effects were maintained following aggregation of observations within participants, indicating that the primary findings were not solely attributable to clustering of repeated observations within individuals. These findings were consistent with the secondary equivalence and variability analyses, which demonstrated clear non-equivalence between placements for total distance and comparatively smaller, threshold-dependent differences for peak speed (Table 3; S1 Text).

## Discussion

The principal findings of the present study were that vertical sensor placement materially influenced the agreement characteristics of GPS-derived total distance and peak-speed measurements. The manufacturer-recommended upper-back placement demonstrated substantially closer agreement with criterion measures, whereas the mid-back placement introduced substantial systematic underestimation of total distance, wider limits of agreement, and greater measurement variability. Placement-related effects differed according to movement metric, with total distance demonstrating substantial placement-dependent bias and poor agreement between placements, whereas peak speed was characterised by comparatively smaller bias but increased variability at higher running speeds. Collectively, these findings indicate that GPS-derived outputs are sensitive to relatively small changes in vertical sensor location and that upper-back and mid-back placements should not be considered interchangeable within applied athlete-monitoring contexts.

### Total-distance agreement characteristics

The upper-back GPS placement demonstrated close agreement with the criterion distance measure during the modified team-sport simulation circuit, with a small mean bias of 0.57 m, narrow limits of agreement, and low measurement variability (CV = 1.54%). These findings are consistent with previous investigations reporting acceptable measurement accuracy of contemporary 10 Hz GPS systems for total-distance assessment during team-sport locomotor activities [4,5,8,9,34]. The observed accuracy may reflect the relatively stable thoracic positioning of the upper-back sensor, which may reduce excessive oscillation and help preserve signal consistency during intermittent locomotor activities [17,21], although these mechanisms were not directly assessed in the present study.

In contrast, the mid-back placement substantially underestimated total distance relative to the criterion measure, producing a mean bias of −12.69 m and considerably greater measurement variability (CV = 4.18%). Direct comparison between placements demonstrated a mean difference of 13.26 m and extremely poor agreement (ICC(A,1) = 0.089; CCC = 0.089), indicating that the two placement locations should not be considered interchangeable for total-distance assessment. These findings demonstrate that a relatively small 10 cm inferior displacement of the sensor materially altered GPS-derived measurement outputs, supporting previous suggestions that wearable tracking outputs may be sensitive to sensor location and body-segment dynamics [17,21,23].

The mechanisms underpinning these placement effects are likely multifactorial. Trunk-mounted wearable systems are sensitive to movement-specific perturbations arising from locomotion, accelerations, impacts, and physical contacts [17,19,21,23]. Lower sensor placement may expose the device to greater soft-tissue movement, rotational artefact, and local trunk-motion variability, although these mechanisms were not directly assessed in the present study. Such effects may influence the signal-processing and velocity-estimation procedures used within GPS algorithms [7,36], and therefore represent one possible explanation for the cumulative distance underestimation observed in the present study.

These findings have important implications for applied practice. GPS-derived total distance is widely used to quantify external load, monitor fatigue responses, evaluate training interventions, and guide return-to-play decision-making within team sports [1,2,37]. The substantial placement-dependent differences observed in the present study indicate that relatively small changes in vertical sensor location can materially influence GPS-derived total-distance measurements independent of true locomotor performance. Consequently, practitioners should standardise sensor-placement procedures wherever possible and avoid comparing datasets derived from different mounting locations where placement consistency cannot be assured. Although the prevalence of placement inconsistency in applied environments remains unclear, the present findings demonstrate that relatively small differences in vertical sensor location can materially influence GPS-derived outputs and may compromise the interchangeability of measurements obtained from different sensor locations. This recommendation aligns with recent reviews emphasising the importance of methodological standardisation when interpreting athlete-monitoring data across applied settings [3,12]. Similar recommendations have been reported within local-positioning-system research, where consistent sensor placement was identified as an important factor influencing measurement reliability and the interpretation of athlete-monitoring data [21].

### Peak-speed agreement characteristics

For peak speed, the upper-back placement again demonstrated stronger agreement with the criterion device than the mid-back placement. Upper-back measurements exhibited a small negative bias (−0.11 m·s^-1^), low variability (CV = 4.09%), and strong agreement with the criterion measure (ICC(A,1) = 0.864; CCC = 0.864). These findings are broadly consistent with previous research reporting acceptable measurement accuracy of contemporary GPS systems for sprint and maximal running velocity assessment under field-based conditions [9,10,34,38,39].

The mid-back placement demonstrated a larger negative bias (−0.27 m·s^-1^), wider limits of agreement, greater measurement variability (CV = 5.22%), and reduced agreement with the criterion measure (ICC(A,1) = 0.770; CCC = 0.769). Although the magnitude of the placement effect was considerably smaller than that observed for total distance, the findings nevertheless indicate that vertical sensor location influences peak-speed measurements, consistent with previous observations that wearable tracking outputs may be sensitive to sensor position and movement-related artefact [17,19,21,23].

The comparatively smaller placement effects observed for peak speed may reflect the discrete nature of maximal velocity detection. One possible explanation is that total-distance measurements accumulate positional error throughout movement, whereas peak-speed measurements are derived from relatively brief periods of maximal velocity and may therefore be less susceptible to cumulative tracking inaccuracies. Nevertheless, visual inspection of the Bland–Altman plots suggested greater dispersion at higher running speeds, indicating that placement-related signal instability remains relevant during high-intensity locomotor activity. Similar velocity-dependent increases in measurement variability have been reported during rapid accelerations, decelerations, and sprint-based activities [7,10,36].

Collectively, these findings suggest that placement effects are metric-specific rather than uniformly expressed across GPS-derived variables. Total distance appeared particularly sensitive to vertical sensor displacement, whereas peak speed demonstrated greater resilience to placement-related changes despite some deterioration in agreement characteristics at higher running velocities. This observation is consistent with previous research indicating that wearable sensor outputs may respond differently to device location and movement dynamics depending on the metric under investigation [21,23].

### Agreement characteristics and interchangeability

A key finding of the present study was the lack of interchangeability between upper-back and mid-back placements. Direct between-placement comparisons demonstrated substantial disagreement for total distance and smaller but meaningful disagreement for peak speed. Importantly, the observed differences were systematic rather than random, indicating that vertical sensor placement influenced measurement behaviour in a consistent manner. Similar observations have been reported in recent measurement-system comparison research, where technologies designed to quantify the same performance construct demonstrated differing levels of practical equivalence and interchangeability despite strong overall associations [30].

The secondary equivalence analyses provided formal statistical support for these observations (Table 3). Upper-back and mid-back placements were not equivalent for total distance under either the primary (±5.0 m) or sensitivity (±8.5 m) equivalence bounds, reinforcing the conclusion that the two placements should not be considered interchangeable for this metric. In contrast, peak-speed equivalence was dependent upon the threshold applied, with equivalence demonstrated under the practical ±0.20 m·s^-1^ criterion but not under the more stringent ±0.10 m·s^-1^ criterion. Supplementary variability analyses further demonstrated substantially greater measurement error for the mid-back placement, particularly for total distance (S1 Text), indicating that the observed placement effects were characterised by both systematic bias and increased measurement variability.

The observed placement effects exceeded the magnitude of typical measurement error reported for contemporary 10 Hz GPS systems [4,8,9], suggesting that the differences are unlikely to reflect normal device variability alone.

This distinction is important because strong associations between measurement methods do not necessarily imply acceptable agreement or interchangeability [15,40]. Recent methodological reviews have emphasised the importance of distinguishing between validity, reliability, agreement, and interchangeability when evaluating wearable athlete-monitoring technologies and have highlighted the limitations of relying solely on correlation-based statistics when assessing measurement performance [24,25,29]. The present findings support these recommendations and demonstrate that apparently minor procedural differences can materially influence GPS-derived outputs. More broadly, the athlete-monitoring literature demonstrates that relatively small methodological differences in system configuration can meaningfully alter measurement outputs and agreement characteristics, whether arising from sensor placement, device configuration, or the underlying tracking technology employed [26,29,41].

The participant-level sensitivity analyses yielded agreement estimates that were highly consistent with the primary observation-level analyses (S2 Text), with similar bias, variability, and agreement characteristics observed across all comparisons. The persistence of the placement effect following participant-level aggregation strengthens confidence that the reported differences represent a genuine characteristic of the measurement system rather than an artefact of the repeated-measures structure of the dataset.

These present findings extend previous work examining the influence of sensor location on wearable tracking outputs [17,21,23] by demonstrating that even relatively small vertical changes in sensor position can materially alter GPS-derived measurements. From an applied perspective, this suggests that sensor placement should be treated as a standardised methodological variable rather than a trivial procedural consideration.

### Practical applications

The present findings have several practical implications for practitioners using GPS technology within team-sport environments. First, the manufacturer-recommended upper-back placement should remain the preferred mounting location when quantifying total distance and peak speed due to its superior agreement characteristics and lower measurement variability. Second, practitioners should avoid interchanging upper-back and mid-back placements within longitudinal monitoring programmes, as placement-related error may confound interpretation of athlete performance and training-load responses. Third, consistent placement procedures should be implemented across training sessions, competitive matches, and rehabilitation monitoring to minimise artificial measurement variation and improve confidence in athlete-monitoring decisions [42].

Although the present investigation was conducted using PlayerTek 10 Hz GPS units, the findings may also have practical relevance for contemporary Catapult One users, as both systems share the same technological platform and employ comparable upper-torso mounting configurations; however, direct evaluation of contemporary Catapult One hardware remains necessary. Nevertheless, further research is required to determine whether similar placement effects exist across newer hardware generations and alternative GPS manufacturers. More broadly, the findings reinforce growing recognition that device configuration and implementation procedures can influence athlete-monitoring outputs independently of underlying locomotor performance [3,12].

### Limitations and future directions

Several limitations should be acknowledged. First, the study utilised a modified team-sport simulation circuit rather than unrestricted match-play conditions, which may limit ecological generalisability to highly dynamic competitive environments. Second, although participant-level sensitivity analyses yielded agreement estimates that were highly consistent with the primary observation-level analyses (S2 Text), the repeated-measures structure of the dataset may nevertheless influence the precision of some estimates. Third, while consistent unit-allocation procedures were employed throughout testing to minimise device-related variability, the practical study design could not completely separate the effects of vertical sensor placement from potential inter-unit variability. Consequently, a small proportion of the observed differences may reflect device-specific measurement characteristics rather than vertical sensor location alone. Nevertheless, the consistency of the observed placement effects across multiple agreement statistics and the participant-level sensitivity analyses strengthens confidence that vertical sensor placement was the most likely explanation for the observed differences.

Because the total-distance criterion was invariant across laps, variance-dependent agreement statistics could not be estimated, and the corresponding Bland–Altman plots exhibited the characteristic linear structure associated with fixed-reference designs. Under these conditions, agreement is most appropriately interpreted using Bland–Altman bias, limits of agreement, typical error (TE), and coefficient of variation (CV), rather than variance-dependent agreement statistics. Fourth, satellite count and dilution-of-precision (DOP) metrics, which are commonly reported as indicators of GPS signal quality, were not available within the proprietary PlayerTek software environment and therefore could not be reported. Consequently, their potential influence on measurement accuracy could not be evaluated and should be considered when interpreting the present findings. In addition, only a single GPS model and sampling frequency were examined; therefore, the present findings may not generalise to alternative hardware systems, processing algorithms, or sensor-fusion approaches. Finally, although the reference measures provided robust standards for total distance and peak speed, additional biomechanical analyses may help clarify the mechanistic basis of the observed placement effects.

Future research should investigate the influence of vertical sensor placement during unrestricted match play and high-intensity multidirectional movement tasks. Further work examining the interaction between sensor location, signal-processing algorithms, inertial-sensor fusion techniques, reference-measure selection, and device generation may improve understanding of placement-related measurement error and agreement characteristics [24,25]. Investigation of alternative anatomical mounting locations and wearable-system configurations may also help establish best-practice recommendations for athlete-monitoring applications. Finally, future work should examine whether calibration or correction procedures can mitigate systematic placement bias and improve comparability between sensor locations within longitudinal athlete-monitoring datasets.

## Conclusion

Within the conditions of the present study, GPS-derived total-distance and peak-speed measurements were sensitive to vertical sensor placement during team-sport locomotor activity. The manufacturer-recommended upper-back placement demonstrated closer agreement with criterion measures, narrower limits of agreement, and lower measurement variability than the mid-back placement. Placement-related effects were metric-specific and particularly pronounced for total distance, where substantial systematic underestimation and poor agreement between placements were observed in the mid-back condition. Peak-speed measurements were comparatively less affected by sensor displacement than total-distance measurements, although agreement remained consistently superior at the upper-back location. These findings indicate that upper-back and mid-back placements should not be considered interchangeable for total-distance assessment and highlight the importance of standardised sensor-placement procedures within applied athlete-monitoring environments.

## Supporting information

S1 Text Secondary variability analysis. Supplementary methods and Supplementary Table S1 presenting variability comparisons based on absolute error relative to criterion measures.
(DOCX)

S2 Text Participant-level sensitivity analysis. Supplementary methods and Supplementary Table S2 presenting participant-level agreement statistics for total distance and peak speed.
(DOCX)

S3 Dataset Anonymised analytical dataset. Anonymised analytical dataset underlying the analyses reported in this study.
(XLSX)
