# Supplementary material for: Influence of vertical GPS sensor placement on criterion agreement and interchangeability of distance and speed measures
Source: PLoS One. 2026 Sep 10;21(9):e0357116. doi: 10.1371/journal.pone.0357116 (PMC13561334; doi:10.1371/journal.pone.0357116)
Supplement: Supplementary file 1 [file pone.0357116.s001.docx]

**S1 Text. Secondary Variability Analysis**

**Supplementary Methods**

Secondary variability analyses were conducted to compare measurement error between upper-back and mid-back GPS placements. Absolute error was calculated relative to the criterion measure for each participant. For total distance, absolute error was calculated relative to the fixed criterion distance (170.40 m). For peak speed, absolute error was calculated relative to the 1080 Sprint reference measure.

Participant-level mean absolute errors were calculated for each placement. Mean paired differences in absolute error between placements were estimated using bootstrap procedures with 10,000 resamples, and corresponding 95% confidence intervals were calculated. Error ratios were calculated as the mean absolute error of the mid-back placement divided by that of the upper-back placement. Variability analyses were performed using participant-level mean values (n = 55 for total distance; n = 68 for peak speed).

**Supplementary Table S1.** Variability comparisons based on absolute error relative to criterion measures

| **Variable** | **Upper-Back Absolute Error** | **Mid-Back Absolute Error** | **Mean Paired Difference** | **Bootstrap 95% CI** | **Error Ratio** |
| --- | --- | --- | --- | --- | --- |
| Total Distance | 2.17 m | 12.96 m | 10.79 m | 8.77–12.92 m | 5.97 |
| Peak Speed | 0.356 m·s⁻¹ | 0.436 m·s⁻¹ | 0.081 m·s⁻¹ | 0.024–0.143 m·s⁻¹ | 1.23 |

Note. Absolute error was calculated relative to the criterion measure for each participant. Mean paired differences represent mid-back minus upper-back absolute errors. Bootstrap confidence intervals were derived using 10,000 resamples. Error ratio = mid-back absolute error ÷ upper-back absolute error. Positive mean paired differences indicate greater measurement error in the mid-back placement relative to the upper-back placement.
