# Supplementary material for: Influence of vertical GPS sensor placement on criterion agreement and interchangeability of distance and speed measures
Source: PLoS One. 2026 Sep 10;21(9):e0357116. doi: 10.1371/journal.pone.0357116 (PMC13561334; doi:10.1371/journal.pone.0357116)
Supplement: Supplementary file 2 [file pone.0357116.s002.docx]

**S2 Text. Participant-level sensitivity analysis.**

**Supplementary Methods**

Participant-level sensitivity analyses were conducted to assess whether the primary findings were influenced by the repeated-measures structure of the dataset. For each participant, repeated observations were aggregated to participant-level means prior to analysis, and agreement statistics were recalculated using these aggregated values. This approach reduces the influence of within-participant clustering and allows evaluation of whether the direction and magnitude of the observed placement effects were maintained following participant-level aggregation.

**Supplementary Table S2.** Participant-level agreement statistics for total distance and peak speed.

| **Metric** | **Comparison** | **n** | **Bias** | **95% LoA** | **TE** | **CV (%)** | **ICC(A,1)** | **CCC** |
| --- | --- | --- | --- | --- | --- | --- | --- | --- |
| Distance | Upper vs Criterion | 55 | 0.53 m | −4.68–5.75 m | 1.88 m | 1.10 | NA | NA |
| Distance | Mid vs Criterion | 55 | −12.96 m | −27.93–2.02 m | 5.40 m | 3.30 | NA | NA |
| Distance | Upper vs Mid | 55 | 13.49 m | −0.09–27.07 m | 4.90 m | 2.98 | 0.071 | 0.071 |
| Speed | Upper vs Reference | 68 | −0.10 m·s⁻¹ | −0.91–0.72 m·s⁻¹ | 0.29 m·s⁻¹ | 3.76 | 0.889 | 0.887 |
| Speed | Mid vs Reference | 68 | −0.24 m·s⁻¹ | −1.24–0.76 m·s⁻¹ | 0.36 m·s⁻¹ | 4.67 | 0.808 | 0.806 |
| Speed | Upper vs Mid | 68 | 0.14 m·s⁻¹ | −0.35–0.64 m·s⁻¹ | 0.18 m·s⁻¹ | 2.31 | 0.939 | 0.938 |

Note. Bias = mean difference between methods. LoA = 95% limits of agreement. TE = typical error. CV = coefficient of variation. CCC = Lin’s concordance correlation coefficient. ICC(A,1) = intraclass correlation coefficient. Participant-level analyses were conducted using participant-level mean values to evaluate the influence of repeated observations on the primary agreement analyses. ICC(A,1) and CCC were not estimable for criterion-distance comparisons because the criterion distance was invariant (170.40 m) across participants, rendering variance-dependent agreement statistics inappropriate.

The participant-level analyses yielded findings that were highly consistent with the primary observation-level analyses. Across both total distance and peak speed, the upper-back placement remained in closer agreement with the criterion/reference measures than the mid-back placement. The direction and magnitude of the observed placement effects were maintained following aggregation of observations within participants, indicating that the primary findings were not solely attributable to clustering of repeated observations within individuals.
