# Supplementary material for: Influence of vertical GPS sensor placement on criterion agreement and interchangeability of distance and speed measures
Source: PLoS One. 2026 Sep 10;21(9):e0357116. doi: 10.1371/journal.pone.0357116 (PMC13561334; doi:10.1371/journal.pone.0357116)
Supplement: Supplementary file 3 [file pone.0357116.s005.docx]

**Response to Reviewers**

**Manuscript ID**

PONE-D-26-29939

**Manuscript Title**

Influence of Vertical GPS Sensor Placement on Criterion Agreement and Interchangeability of Distance and Speed Measures

**Response to the Academic Editor and Reviewer**

We sincerely thank the Academic Editor and Reviewer #1 for their careful evaluation of our manuscript and for their constructive comments and suggestions. We have considered each point in detail and believe that the revisions have substantially strengthened the manuscript.

In preparing the revised submission, we undertook a comprehensive revision of the manuscript. The principal changes include clarification of the statistical methodology; additional participant-level sensitivity, variability, and equivalence analyses; expanded discussion of methodological limitations; refinement of the interpretation of the agreement analyses; standardisation of statistical terminology and notation throughout the manuscript; preparation and public deposition of an anonymised analytical dataset; and revision of the Data Availability Statement in accordance with **PLOS ONE** policy.

For clarity, each comment is reproduced in **bold**, followed by our response and a summary of the corresponding revisions made to the manuscript.

**Note:** Unless otherwise stated, all page and line references in this response correspond to the **tracked-changes version** of the revised manuscript submitted alongside the clean manuscript. These references are provided to facilitate identification of the revisions.

**Response to the Academic Editor**

**Academic Editor Comment 1**

*“Please ensure that your manuscript meets PLOS ONE's style requirements, including those for file naming.”*

**Response**

We thank the Academic Editor for highlighting this requirement. The manuscript has been reviewed and revised to ensure full compliance with the PLOS ONE style requirements. File naming, manuscript formatting, tables, figures, supporting information, and submission files have all been checked against the current PLOS ONE author guidelines and formatting templates.

**Changes made**

- Reviewed the manuscript formatting against the current PLOS ONE author guidelines and formatting requirements.
- Checked file naming conventions for all submission files.
- Reviewed tables, figures, and Supporting Information for consistency with PLOS ONE requirements.
- Verified the formatting and organisation of all submission files prior to resubmission.

**Academic Editor Comment 2**

*“In the online submission form you indicate that your data is not available for proprietary reasons and have provided a contact point for accessing this data. Please note that your current contact point is a co-author on this manuscript. According to our Data Policy, the contact point must not be an author on the manuscript and must be an institutional contact, ideally not an individual. Please revise your data statement to a non-author institutional point of contact, such as a data access or ethics committee, and send this to us via return email. Please also include contact information for the third party organization, and please include the full citation of where the data can be found.”*

**Response**

We thank the Academic Editor for highlighting this important issue. In response, we have revised the manuscript's Data Availability Statement to comply with the PLOS ONE Data Availability Policy. An anonymised analytical dataset containing the participant-level data underlying the analyses reported in the manuscript has been provided as S2 File with this revised submission and has also been deposited in the Ulster University Research Portal (PURE) public repository, where it has been assigned a persistent DOI (<https://doi.org/10.21251/a38ba55b-963f-4408-ba82-2993fda0614b>) and released under a CC BY 4.0 licence. The Data Availability Statement has been updated to provide the repository citation and DOI. Throughout this process, participant confidentiality and institutional data protection requirements have been maintained.

**Changes made**

- **Supporting Information:** Prepared and provided an anonymised analytical dataset containing the participant-level data underlying the reported analyses as **S2 File.**
- **Repository deposition:** Deposited the anonymised analytical dataset in the Ulster University Research Portal (PURE) and assigned the persistent DOI: <https://doi.org/10.21251/a38ba55b-963f-4408-ba82-2993fda0614b> (CC BY 4.0).
- **Data Availability** Revised to provide the repository citation and DOI.

**Academic Editor Comment 3**

*“Your ethics statement should only appear in the Methods section of your manuscript. If your ethics statement is written in any section besides the Methods, please delete it from any other section.”*

**Response**

We thank the Academic Editor for this observation. The manuscript has been revised so that all ethics-related information is presented exclusively within the Methods section. The duplicate ethics statements that previously appeared elsewhere in the manuscript have been removed, and the ethics information within the Methods has been consolidated to improve clarity and avoid duplication.

**Changes made**

- **Page 8, lines 176–177**: Revised the Participants subsection to remove the duplicated informed consent statement, leaving only: *"All participants were free from injury at the time of testing."*
- **Page 8, lines 179–182**: Consolidated the ethics information into a single statement within the Methods: "Ethical approval for this study was granted by the Ulster University Research Ethics Committee. All participants provided written informed consent prior to participation, and all study procedures were conducted in accordance with the principles of the Declaration of Helsinki."
- **Page 35, lines 741–743:** Removed the duplicate **Ethics Approval** section that previously appeared outside the Methods section.
- **Page 35, lines 745–746:** Removed the duplicate **Consent to Participate** section that previously appeared outside the Methods section.

**Academic Editor Comment 4**

*“Please include captions for your Supporting Information files at the end of your manuscript, and update any in-text citations to match accordingly.”*

**Response**

We thank the Academic Editor for identifying this omission. A **Supporting Information** section has now been added to the end of the manuscript. Descriptive captions have been provided for both submitted Supporting Information files (**S1 File** and **S2 File**), and the manuscript has been reviewed to ensure that all in-text references to the Supporting Information are consistent with the submitted files.

**Changes made**

- **Page 36, lines 758–764:** Added a **Supporting Information** section at the end of the manuscript.
- Added the following captions:
  - **S1 File. Supplementary Materials.** Supplementary methods describing the secondary variability analysis and participant-level sensitivity analysis, together with **Supplementary Tables S1 and S2** containing additional agreement and variability analyses supporting the findings of the main manuscript.
  - **S2 File. Anonymised analytical dataset.** Dataset underlying the analyses reported in this study.
- Reviewed and updated in-text references to the supplementary material where necessary.
- Verified consistency between the manuscript and the submitted Supporting Information files.

**Academic Editor Comment 5**

*“If the reviewer comments include a recommendation to cite specific previously published works, please review and evaluate these publications to determine whether they are relevant and should be cited. There is no requirement to cite these works unless the editor has indicated otherwise.”*

**Response**

We thank the Academic Editor for this recommendation. We carefully reviewed the reviewer comments and confirmed that no specific previously published works were recommended for citation. Nevertheless, the literature cited in the revised manuscript was reviewed to ensure that it appropriately reflects the current evidence relevant to the objectives, methodology, and interpretation of the present study.

**Changes made**

- Reviewed the reviewer comments to determine whether any additional publications had been recommended for citation.
- Reviewed the cited literature during manuscript revision to ensure that the references appropriately support the revised manuscript and reflect the current evidence base.

**Response to Reviewer #1**

We thank the reviewer for their thorough and constructive assessment of our manuscript. We appreciate the positive assessment that the manuscript is largely technically sound and written in clear English. In response to the reviewer's comments, we have strengthened the manuscript by:

- clarifying the availability of satellite signal quality metrics (DOP and satellite count);
- expanding the statistical methodology to address repeated-measures clustering and the interpretation of Bland–Altman analyses for a fixed-reference criterion;
- preparing and publicly depositing an anonymised analytical dataset in the **Ulster University Research Portal (PURE)** and revising the **Data Availability Statement** in accordance with **PLOS ONE** requirements; and
- undertaking a comprehensive editorial review to improve the consistency of statistical terminology, notation, and formatting throughout the manuscript.

**Reviewer #1**

**Reviewer comment 1A**

*"The manuscript is largely sound, but I have selected 'partly' due to the lack of satellite signal quality metrics (DOP and satellite count), which are critical for GPS validity."*

**Response**

We thank the reviewer for this constructive observation. We acknowledge that satellite count and dilution-of-precision (DOP) metrics are commonly reported as indicators of GPS signal quality. Unfortunately, these variables were not available within the proprietary PlayerTek software environment and therefore could not be reported. To address this limitation, we have clarified this point in the **Methods** section and expanded the **Limitations and Future Directions** section to acknowledge that the absence of these metrics represents a limitation of the proprietary software environment and should be considered when interpreting the present findings.

**Changes made**

- **Page 13, lines 266-267:** Clarified that satellite count and dilution-of-precision (DOP) metrics were not available within the proprietary PlayerTek software environment and therefore could not be reported.
- **Page 33, lines 693-700:** Expanded the **Limitations and Future Directions** section to acknowledge that satellite count and DOP metrics were unavailable within the proprietary PlayerTek software environment, that their potential influence on measurement accuracy could not be evaluated, and that this should be considered when interpreting the present findings.

**Reviewer Comment 1B**

*"Additionally, the potential for inter-unit variability was not fully controlled for every participant, which introduces a minor limitation in isolating the effect of sensor placement."*

**Response**

We thank the reviewer for this helpful observation. We agree that, although consistent unit-allocation procedures were employed throughout testing to minimise device-related variability, the practical study design could not completely separate the effects of vertical sensor placement from potential inter-unit variability. To acknowledge this more explicitly, we have strengthened the Limitations and Future Directions section by clarifying that a small proportion of the observed differences may reflect device-specific measurement characteristics rather than vertical sensor placement alone.

**Changes made**

- **Page 32, lines 671–679:** Strengthened the discussion of inter-unit variability in the **Limitations and Future Directions** section.
- Clarified that consistent unit-allocation procedures were employed throughout testing to minimise device-related variability.
- Clarified that, although complete separation of vertical sensor placement effects from inter-unit variability was not possible within the practical study design, the consistency of the agreement analyses and participant-level sensitivity analyses supports the interpretation that vertical sensor placement was the most likely explanation for the observed differences.

**Reviewer Comment 2A**

"...the authors need to clarify if the clustering effect of repeated measures was fully addressed in their models."

**Response**

We thank the reviewer for this important observation. We agree that the repeated-measures structure of the dataset required explicit consideration. In response, we expanded the **Statistical Analysis** section to clarify that participant-level sensitivity analyses were undertaken by aggregating repeated observations to participant-level mean values before recalculating the agreement statistics. We also clarified the corresponding findings in the **Results** and **Discussion**, demonstrating that the participant-level analyses yielded agreement estimates that were highly consistent with the primary observation-level analyses, indicating that the reported placement effects were robust to the repeated-measures structure of the dataset.

**Changes made**

- **Statistical Analysis (Page 16 lines 329–335):** Expanded the **Statistical Analysis** section to describe the participant-level sensitivity analyses undertaken to assess the robustness of the primary findings to clustering of repeated observations within individuals.
- **Results:** Clarified that the participant-level sensitivity analyses yielded findings that were highly consistent with the primary observation-level analyses (**Supplementary Table S2**).
- **Discussion (Page 32, lines 674-679):** Expanded the interpretation to explain that the consistency of the participant-level sensitivity analyses strengthens confidence that the reported placement effects were not solely attributable to clustering of repeated observations within participants.

**Reviewer Comment 2B**

*"Furthermore, the implications of the fixed-reference distance on the Bland–Altman LoA interpretation require a more nuanced statistical justification to ensure the analysis is entirely robust."*

**Response**

We thank the reviewer for this helpful observation. We agree that the fixed-reference design used for the total-distance protocol warranted additional statistical clarification. The original manuscript explained why variance-dependent agreement statistics (**ICC(A,1)** and **CCC**) were not appropriate for criterion-distance comparisons when the reference distance remained invariant across observations. In response to the reviewer's comment, we have strengthened the **Statistical Analysis** section by explicitly clarifying that, under these conditions, agreement is most appropriately interpreted using Bland–Altman bias, limits of agreement (LoA), typical error (TE), and coefficient of variation (CV). We have also refined the **Discussion** to explain the implications of the fixed-reference design for interpreting the Bland–Altman analyses and the resulting agreement statistics.

**Changes made**

- **Statistical Analysis (Page 15, lines 304–307):** Expanded the **Statistical Analysis** section to clarify that, because the total-distance criterion remained invariant across observations, agreement was interpreted primarily using Bland–Altman bias, limits of agreement (LoA), typical error (TE), and coefficient of variation (CV), which remain appropriate for evaluating agreement against a fixed-reference criterion.
- **Discussion – Limitations and Future Directions (Pages 32-33, lines 684–689):** xpanded the **Discussion** to explain the implications of the fixed-reference design for interpreting the Bland–Altman analyses and to clarify why Bland–Altman bias, limits of agreement (LoA), typical error (TE), and coefficient of variation (CV) provide the most appropriate basis for interpreting agreement when the criterion measure is invariant.

**Reviewer Comment 3**

*"...the raw data points...should upload the raw dataset..."*

**Response**

We thank the reviewer for highlighting the importance of compliance with the PLOS ONE Data Availability Policy. In response, we have prepared an anonymised analytical dataset containing the participant-level data underlying the analyses reported in the manuscript. To ensure that the underlying data are immediately available for editorial assessment and to comply fully with the PLOS ONE Data Availability Policy, the anonymised analytical dataset has been included as **S2 File** with this revised submission. The dataset has also been deposited in the **Ulster University Research Portal (PURE)**, where it has been assigned a persistent DOI (<https://doi.org/10.21251/a38ba55b-963f-4408-ba82-2993fda0614b>) and released under a CC BY 4.0 licence. The manuscript's Data Availability Statement has been updated accordingly. Throughout this process, participant confidentiality and institutional data protection requirements have been maintained.

**Changes made**

- **Supporting Information (Page 36, line 758-764):** Prepared and provided an anonymised analytical dataset containing the participant-level data underlying the reported analyses as **S2 File**.
- **Repository deposition:** Deposited the anonymised analytical dataset in the **Ulster University Research Portal (PURE)** and assigned the persistent DOI: <https://doi.org/10.21251/a38ba55b-963f-4408-ba82-2993fda0614b> (CC BY 4.0).
- **Data Availability Statement (Page 34, line 733-736):** Revised to provide the repository citation and DOI.

**Reviewer Comment 4**

"The manuscript is written in clear, intelligible English. While the overall quality is good, I recommend a final proofreading to ensure consistency in technical terminology throughout the manuscript, particularly regarding the formatting of statistical symbols (CV, ICC, CCC)."

**Response**

We thank the reviewer for this positive assessment of the manuscript's clarity and presentation. In response to this recommendation, we undertook a comprehensive editorial proofread of the revised manuscript and Supporting Information (**S1 File** and **S2 File**) to improve the consistency of technical terminology, statistical notation, abbreviations, grammar, punctuation, and formatting throughout. Particular attention was given to the consistent presentation of statistical terminology and abbreviations, including coefficient of variation (CV), intraclass correlation coefficient (ICC(A,1)), Lin's concordance correlation coefficient (CCC), typical error (TE), confidence intervals (CI), and limits of agreement (LoA). Statistical notation, table headings, figure captions, and Supporting Information were reviewed to ensure consistent presentation throughout the revised submission.

**Changes made**

- **Manuscript and Supporting Information:** Undertook a comprehensive editorial proofread to improve the consistency of technical terminology, statistical notation, abbreviations, grammar, punctuation, and formatting throughout.
- Standardised the presentation of statistical terminology and abbreviations throughout the manuscript and Supporting Information, including ICC(A,1), CCC, CV, CV (%) (table columns), TE, CI, and LoA.
- Reviewed tables, figures, figure captions, and Supporting Information to ensure consistent terminology, notation, and formatting throughout the revised submission.
- Corrected minor editorial inconsistencies identified during revision.
